# Effectiveness of risk minimization measures for the use of cilostazol in United Kingdom, Spain, Sweden, and Germany

**DOI:** 10.1002/pds.4584

**Published:** 2018-07-25

**Authors:** Jordi Castellsague, Beatriz Poblador‐Plou, Maria Giner‐Soriano, Marie Linder, Oliver Scholle, Brian Calingaert, Christine Bui, Alejandro Arana, Clara Laguna, Francisca Gonzalez‐Rubio, Albert Roso‐Llorach, Alexandra Prados‐Torres, Susana Perez‐Gutthann

**Affiliations:** ^1^ Epidemiology RTI Health Solutions Barcelona Spain; ^2^ Aragon Health Sciences Institute (IACS), IIS Aragon, Hospital Universitario Miguel Servet University of Zaragoza Zaragoza Spain; ^3^ Institut Universitari d'Investigació en Atenció Primària Jordi Gol (IDIAP Jordi Gol) Barcelona Spain; ^4^ Universitat Autònoma de Barcelona Bellaterra Cerdanyola del Vallès Spain; ^5^ Centre for Pharmacoepidemiology, Unit of Clinical Epidemiology, Department of Medicine Karolinska Institutet Stockholm Sweden; ^6^ Department of Clinical Epidemiology Leibniz Institute for Prevention Research and Epidemiology—BIPS Bremen Germany; ^7^ Epidemiology RTI Health Solutions Research Triangle Park NC USA

**Keywords:** cilostazol, database study, intermittent claudication, peripheral artery disease, pharmacoepidemiology, risk minimization

## Abstract

**Purpose:**

The purpose of the study is to evaluate the effectiveness of risk minimization measures—labeling changes and communication to health care professionals—recommended by the European Medicines Agency for use of cilostazol for the treatment of intermittent claudication in Europe.

**Methods:**

Observational study of cilostazol in The Health Improvement Network (United Kingdom), EpiChron Cohort (Spain), SIDIAP (Spain), Swedish National Databases, and GePaRD (Germany).

Among new users of cilostazol, we compared the prevalence of conditions targeted by the risk minimization measures in the periods before (2002‐2012) and after (2014) implementation. Conditions evaluated were prevalence of smoking, cardiovascular conditions, concurrent use of ≥2 antiplatelet agents, concurrent use of potent CYP3A4/CYP2C19 inhibitors and high‐dose cilostazol, early monitoring of all users, and continuous monitoring of users at high cardiovascular risk.

**Results:**

We included 22 593 and 1821 new users of cilostazol before and after implementation of risk minimization measures, respectively. After implementation, the frequency of several conditions related to the labeling changes improved in all the study populations: prevalence of use decreased between 13% (EpiChron) and 57% (SIDIAP), frequency of cardiovascular contraindications decreased between 8% (GePaRD) and 84% (EpiChron), and concurrent use of high‐dose cilostazol and potent CYP3A4/CYP2C19 inhibitors decreased between 6% (Sweden) and 100% (EpiChron). The frequency of other conditions improved in most study populations, except smoking, which decreased only in EpiChron (48% reduction).

**Conclusions:**

This study indicates that the risk minimization measures implemented by the EMA for the use of cilostazol have been effective in all European countries studied, except for smoking cessation before initiating cilostazol, which remains an area of improvement.

**KEY POINTS**

- This study evaluated the effectiveness of risk minimization measures among new users of cilostazol in the United Kingdom, Spain, Sweden, and Germany.
- The observed decrease in the prevalence of cilostazol use, cardiovascular contraindications, and concurrent use of 2 or more antiplatelet drugs or interacting medications indicates that the risk minimization measures were effective in all the study populations.
- Stopping smoking before initiating cilostazol remains an area of improvement, as prevalence of smoking after risk minimization measures decreased in only 1 of 4 study populations where smoking was evaluated.

## 1 INTRODUCTION

Cilostazol is a platelet aggregation inhibitor approved in Europe in 2002 to improve walking distances in patients with intermittent claudication. Cilostazol has been associated with spontaneous reports of serious bleeding and cardiovascular effects including heart attacks, angina, and arrhythmias. The European Medicines Agency (EMA) evaluated the benefits and risks of cilostazol in a referral and recommended implementation of risk minimization measures to restrict the use of cilostazol to patients that could benefit from treatment and in which important risks are minimized.^1^ Risk minimization measures included labeling changes (Table 1) and educational communications directed to health care professionals through the Otsuka Europe website and “Dear Doctor” letters implemented in 2013.

**Table 1:** Cilostazol labeling changes and study variables

Labeling Section	Labeling Changes	Study Variable
Indication	Second‐line use after lifestyle modifications, including smoking cessation and (supervised) exercise programs, failed to sufficiently improve symptoms	Prevalence of current smoking at the start date Second‐line use and supervised exercise were not evaluated
	Physician reassessment of patients after 3 months of treatment with a view to discontinuing cilostazol where an inadequate effect is observed	Visit to the general practitioner or specialist (cardiologist, vascular specialist, or diabetologist) between 2 and 4 months after the start date
		Visit related to intermittent claudication
		Discontinuation within 3 months of treatment
New contraindications	Unstable angina pectoris, myocardial infarction within the last 6 months, or a coronary intervention in the last 6 months	Prevalence of new contraindications before the start date
	Concomitant treatment with 2 or more additional antiplatelet agents (eg, aspirin, clopidogrel)	Concurrent use at the start date or use of 2 or more antiplatelet agents during continuous use of cilostazol
Old contraindications^a^	Severe renal impairment, moderate to severe hepatic impairment, congestive heart failure, predisposing factors for bleeding (active peptic ulcer, hemorrhagic stroke within the prior 6 months, proliferative diabetic retinopathy, and poorly controlled hypertension)	Prevalence of old contraindications before the start date
Warnings and precautions	Close monitoring of patients at increased risk for serious cardiac adverse events as a result of increased heart rate, eg, patients with stable coronary disease or a history of tachyarrhythmias	Rates of visits to general practitioner or specialist during continuous use of cilostazol in patients at increased and not increased risk of cardiac adverse events
Posology	Reduction of the dose to 50 mg twice daily in patients receiving medicines that strongly inhibit CYP3A4 or CYP2C19	Prevalence of concurrent use of high‐dose cilostazol and CYP3A4 or CYP2C19 potent inhibitors, and percentage of concurrent users with reduction of high dose

a Contraindications already included in the labeling of cilostazol before new labeling was recommended by the European Medicines Agency.

To evaluate the impact of these risk minimization measures, we compared the prevalence of cilostazol use and of the conditions targeted by these risk minimization measures before and after these measures were implemented.

## 2 METHODS

### 2.1 Data sources

The study was conducted in The Health Improvement Network (THIN), UK^2^, ^3^, ^4^; the EpiChron cohort (EpiChron) from the Aragon Institute of Health Sciences (IACS), Aragon, Spain^5^, ^6^; the Information System for Research in Primary Care (SIDIAP), Catalonia, Spain^7^; the Swedish National Registers^8^, ^9^; and the German Pharmacoepidemiological Research Database (GePaRD).^10^ The main features of the study databases are presented in Table S1, online supporting information. The baseline characteristics of users of cilostazol before implementation of risk minimization measures have been published elsewhere.^11^

### 2.2 Study population

**Figure 1:**
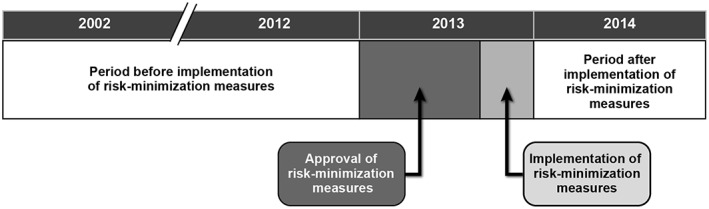
Study timeline in relation to the implementation of risk minimization measures

New users of cilostazol were identified before and after implementation of risk minimization measures (Figure 1). The period before implementation was from the date cilostazol became available in each country through September 14, 2012 in THIN; December 31, 2012 in EpiChron, SIDIAP, and Sweden; and December 31, 2011 in GePaRD. Data for the year 2012 were not available in GePaRD at the time of the baseline assessment.^11^ The period after implementation was the year 2014. New users were defined as patients who received a first‐ever prescription of cilostazol during each study period after having at least 6 months of continuous enrollment in the database. The date of the first cilostazol prescription was defined as the start date. Patients with a recorded prescription of cilostazol at any time before the start date were excluded. New users were followed from the start date until the first of the following: end of enrollment in the database, death, or end of the study period.

### 2.3 Evaluation of the effectiveness of risk minimization measures

We compared the prevalence of new users of cilostazol and the frequency of conditions targeted by risk minimization measures included in the labeling (Table 1) before (2002‐2012) and after (2014) the risk minimization measures were implemented in 2013 (Figure 1). Table 1 describes the labeling changes and the conditions evaluated to assess their impact. These conditions were prevalence of smoking cessation, frequency of early monitoring of patients taking cilostazol, frequency of patients with early discontinuation of cilostazol, frequency of cardiovascular conditions that were new contraindications, frequency of concurrent use of 2 or more antiplatelet agents, increase in monitoring for users at increased risk of serious cardiac events, and frequency of concurrent use of high‐dose cilostazol and potent inhibitors of CYP3A4 or CYP2C19.^12^, ^13^ Information on smoking was available in THIN, EpiChron, and SIDIAP. In Sweden, we evaluated smoking using diagnosis codes for smoking‐related disease and use of smoking‐cessation drugs. Early monitoring of users was assessed by the number of patients who had at least 1 visit to a specialist (vascular surgery, cardiology, diabetology) 2 to 4 months after the start date. These visits were classified as related to peripheral arteriopathy. In Sweden, evaluation of visits was restricted to hospitals and hospital outpatient clinics. In GePaRD, diagnoses are recorded on a quarterly basis, and visits were evaluated by the number of patients who had at least 1 diagnosis for intermittent claudication recorded in the 3 months following the quarter in which cilostazol was started. Early discontinuation of cilostazol was defined as discontinuation occurring within the first 3 months of treatment. New cardiovascular contraindications were unstable angina pectoris and recent myocardial infarction or coronary intervention. We also evaluated the prevalence of contraindications in the labeling before implementation of labeling changes (ie, old contraindications): severe renal impairment, moderate to severe hepatic impairment, congestive heart failure, predisposing factors for bleeding (active peptic ulcer, hemorrhagic stroke within the prior 6 months, proliferative diabetic retinopathy, and poorly controlled hypertension), and history of specific arrhythmias. Concurrent use of cilostazol and 2 or more antiplatelet agents was defined as overlaps of the intended duration of prescriptions of each medication. Monitoring of patients at increased risk of serious cardiac events was evaluated by comparing rates of visits to general practitioners or specialists between patients with and without a history of arrhythmias, hypotension, or coronary heart disease during continuous use of cilostazol. In GePaRD, monitoring was expressed as the number of diagnoses per patient‐year of continuous use, because only the first visit to the same physician is recorded during a quarter. Continuous use of cilostazol was defined as the total number of days covered by all periods of consecutive prescriptions, allowing for a maximum 60‐day gap. Concurrent use of high‐dose cilostazol and potent inhibitors of CYP3A4 or CYP2C19 was defined at the start date and during follow‐up. Concurrent use at the start date was defined as having a prescription for an interacting medication within 3 months before the start date of high‐dose cilostazol. Concurrent use during follow‐up was defined as having a prescription for an interacting medication during the periods of continuous use of high‐dose cilostazol. In THIN and EpiChron, daily dose of cilostazol was calculated from strength of product, package quantity, and dosage instructions. In Sweden, daily dose was calculated assuming a twice‐daily dosage based on the results of a manual review of free text associated with dispensings. In GePaRD, a twice‐daily dosage was also assumed. In SIDIAP, evaluation of daily dose was not conducted, as information on dosage instructions was not available. Medical diagnoses and use of medications were ascertained through the coding system specific to each database (Table S1, online supporting information).

### 2.4 Analysis

The annual prevalence of cilostazol use was calculated in each database as the ratio between the number of cilostazol users (prevalent and new users) in a specific year and the database population. The cumulative proportion of patients discontinuing cilostazol was calculated using survival analysis. Rates of visits were calculated as the number of visits per 100 person‐years of continuous use of cilostazol, except in GePaRD, where the number of diagnoses per patient‐year was used. Crude incidence rate ratios and 95% confidence intervals (CIs) were estimated to compare rates of visits between patients at high risk of cardiac events and patients not at high risk.

At RTI‐HS (THIN data), SIDIAP, Sweden, and GePaRD, analyses were conducted using SAS version 9.3 or 9.4 (Cary, NC: SAS Institute Inc.). Stata v13.0 (StataCorp, 2013) was used in the EpiChron cohort. Stata v13.1 and R 3.1 (R Core Team, 2013) were also used in SIDIAP.

The study protocol was approved by the RTI International institutional review board; ethics committees for THIN, EpiChron, IDIAP, and Sweden; and the statutory health insurance providers and German Federal Insurance Authority in Germany. The protocol was approved by the EMA and posted in the EU PAS Register in March 2013 (EU PAS ID: 3596).^14^

## 3 RESULTS

### 3.1 Prevalence and patterns of use

**Figure 2:**
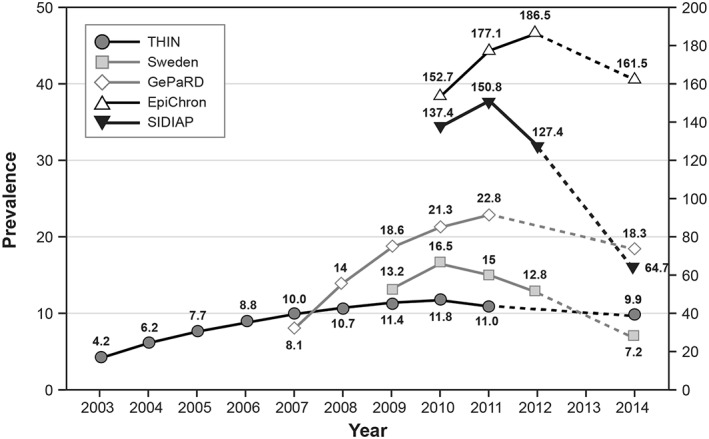
Annual prevalence of use of cilostazol before and after the implementation of risk minimization measures (per 100 000 population). EpiChron, EpiChron cohort from Aragon Health Sciences Institute (IACS), Aragon, Spain; GePaRD, German Pharmacoepidemiological Research Database; SIDIAP, Information System for the Improvement of Research in Primary Care Database, Catalonia, Spain; THIN, The Health Improvement Network. Prevalence was not estimated for 2013, the year of implementation of risk minimization measures

We included 22 593 and 1821 new users of cilostazol before and after implementation of risk minimization measures, respectively (Table 2). EpiChron and SIDIAP contributed the largest number of users in both periods. The annual prevalence of use of cilostazol decreased after 2011 to 2012 in all study populations (Figure 2). The reduction in annual prevalence was calculated by comparing the period after implementation of risk minimization measures to the year with the maximum prevalence before implementation of risk minimization measures. The reduction ranged from −16.1% in THIN to −57.1% in SIDIAP. There was a slightly higher proportion of men than women in all study populations in both periods (Table 2). After implementation of risk minimization measures, the median age decreased in men and women in EpiChron, SIDIAP, and Sweden. The median age in women also decreased in GePaRD. The proportion of users prescribed a daily dose of 200 mg decreased after implementation of risk minimization measures in all study populations except Sweden. Discontinuation of cilostazol at 3 months and at 6 months after the start of treatment increased after implementation of risk minimization measures in THIN, SIDIAP, and Sweden; decreased in EpiChron; and practically did not change in GePaRD.

**Table 2:** Characteristics and patterns of use in new users of cilostazol before and after the implementation of risk minimization measures

Characteristic	THIN UK	EpiChron Aragon Spain	SIDIAP Catalonia Spain	Sweden	GePaRD Germany
Study period					
Before	2002‐2012	2009‐2012	2009‐2012	2008‐2012	2007‐2011
After	2014	2014	2014	2014	2014
Number of users					
Before	1,528	4,024	10,142	2,887	4,012
After	104	367	771	149	430
Men (%)					
Before	65.6	72.2	77.3	52.3	73.3
After	66.3	85.6	78.5	58.4	70.9
Median age before/after (years)					
Men	68.0/69.0	69.0/65.9	68.0/65.0	72.4/69.7	69.0/70.0
Women	71.0/74.0	73.9/69.7	75.0/68.0	75.0/72.5	70.0/69.0
Daily dose 200 mg (%)					
Before	85.7	77.3	NA	78.1	87.9
After	31.7	7.1	NA	79.9	77.0
Discontinuation before/after (%)					
<1 month	28.7/38.5	33.9/25.5	22.2/20.5	38.3/43.0	39.4/40.7
<3 months	52.9/64.4	51.9/30.4	40.6/58.1	39.4/47.9	51.9/52.8
<6 months	62.2/70.3	60.5/35.2	50.4/77.3	65.2/70.6	64.9/68.6
<12 months	71.3/70.3	69.1/45.8	64.6/100.0	81.9/82.6	77.8/77.5

The terms *before* and *after* refer to the periods before and after the implementation of risk minimization measures.

EpiChron, EpiChron cohort from the Aragon Health Sciences Institute (IACS); GePaRD, German Pharmacoepidemiological Research Database; NA, not available; SIDIAP, Information System for the Improvement of Research in Primary Care; THIN, The Health Improvement Network; UK, United Kingdom.

### 3.2 Comorbidity and comedications

The most frequent comorbidities and comedications before and after implementation of risk minimization measures are presented in Tables S2 and S3, online supporting information. The patterns of comorbidities and comedications remained similar before and after implementation of risk minimization measures. Cardiovascular disease was the most frequent comorbidity in all study populations in both periods, followed by diabetes, skin disorders, renal diseases, and bleeding disorders. Antihypertensives, lipid‐modifying agents, platelet aggregation inhibitors, statins, and proton pump inhibitors were the most frequent comedications.

Most cilostazol users were treated concurrently with interacting medications in both periods; however, in EpiChron, SIDIAP, and Sweden, concurrent treatment decreased after the risk minimization measures were implemented. Concurrent use of cilostazol and potent inhibitors of CYP3A4 or CYP2C19 also decreased in all databases. The decrease ranged from 2.7% (Sweden) to 22.3% (THIN) before risk minimization and from 0.7% (Sweden) to 17.3% (THIN) after risk minimization (Table S4, online supporting information).

### 3.3 Evaluation of the effectiveness of risk minimization measures

**Figure 3:**
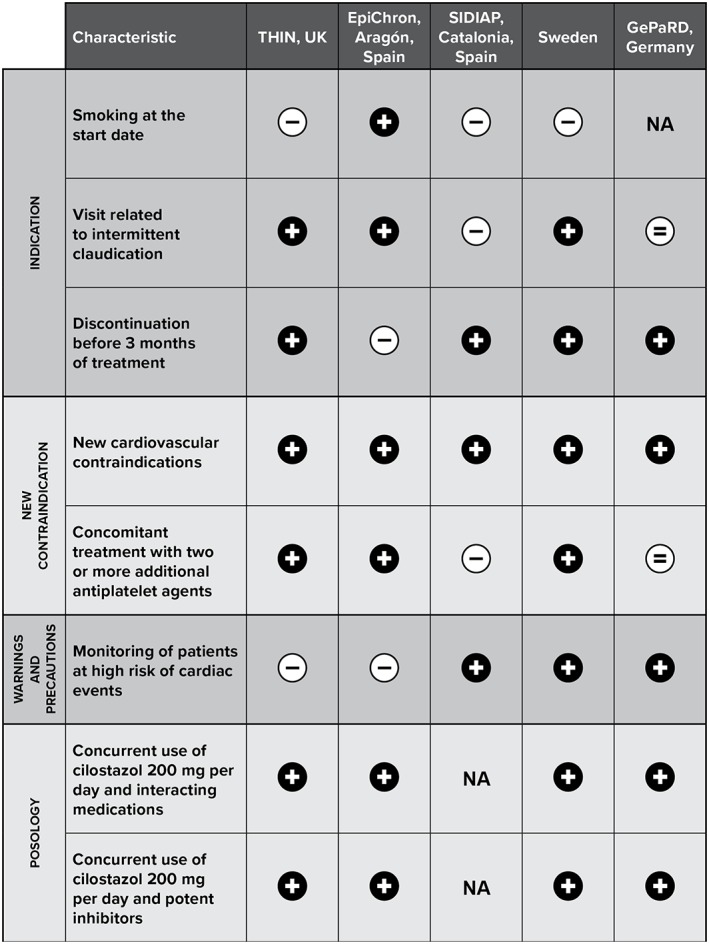
Summary of improvement in or worsening of characteristics impacted by risk minimization measures, by data source. GePaRD, German Pharmacoepidemiological Research Database; NA, not available; SIDIAP, Information System for the Improvement of Research in Primary Care Database; THIN, The Health Improvement Network; UK, United Kingdom. Classification was based on a 5% change from before to after the implementation of risk minimization measures. Values below 5% were considered to represent no change. + = improvement after the labeling changes; − = worsening after the labeling changes; equal = no changes after the labeling changes

Results of the assessment of the risk minimization measures are presented in Table 3 and Figures 2 and 3. After implementation of risk minimization measures, the parameters that improved in all study populations were the prevalence of new cardiovascular contraindications and the concurrent use of cilostazol 200 mg per day and interacting medications, including potent inhibitors of CYP3A4 or CYP2C19. Discontinuation of cilostazol within the first 3 months of treatment, concurrent use with 2 or more antiplatelet agents, and monitoring of patients at high risk of cardiac events improved in at least 3 of the study populations. The prevalence of current smoking at the start date decreased only in EpiChron. Overall, Sweden, followed by THIN and EpiChron, were the study populations with the highest number of parameters improved after implementation of labeling changes (Figure 2). We also evaluated the prevalence of old contraindications (in the labeling before labeling changes) before and after implementation of risk minimization measures (Table S5, online supporting information). After labeling changes, the prevalence of old contraindications decreased in THIN (10.0% before, 8.7% after) and EpiChron (6.2%, 5.5%), increased in SIDIAP (39.1%, 51.5%) and GePaRD (51.8%, 54.7%), and was the same as before labeling changes in Sweden (12.2%, 12.1%).

**Table 3:** Assessment of labeling changes before and after the implementation of risk minimization measures

Labeling Change	THIN UK	EpiChron Aragon Spain	SIDIAP Catalonia Spain	Sweden	GePaRD Germany
Indication					
Second‐line use after lifestyle modifications, including smoking cessation					
Smoking (%)^a^					
Before	30.4	15.9	32.3	3.2	NA
After	37.5	8.2	45.5	4.0	NA
Physician reassessment of patients after 3 months of treatment with a view to discontinuing cilostazol where an inadequate effect is observed					
Early monitoring (%)^b^					
Before	49.6	21.3	53.5	8.5	62.2
After	69.2	24.2	10.8	13.0	63.0
Early discontinuation (%)^c^					
Before	52.9	51.9	40.6	39.4	50.3
After	64.4	30.4	58.1	47.9	52.8
New contraindications					
New cardiovascular contraindications (%)^d^					
Before	1.5	1.7	3.0	5.2	11.6
After	1.0	0.3	0.9	2.7	10.7
Concurrent treatment with ≥2 antiplatelet agents (%)					
Before	9.8	13.5	6.3	8.4	7.5
After	2.9	7.4	6.7	6.7	7.7
Warnings and precautions					
Monitoring of patients at high risk of cardiac events (RR, 95% CI)^e^					
Before	1.08 (1.05‐1.10)	1.12 (1.10‐1.13)	1.19 (1.17‐1.22)	1.90 (1.84‐1.97)	1.03 (0.99‐1.08)
After	0.88 (0.71‐1.09)	0.97 (0.90‐1.05)	1.75 (1.63‐1.88)	2.08 (1.65‐2.64)	1.24 (0.99‐1.56)
Posology					
Concurrent use of cilostazol 200 mg and interacting medications (%)					
Before	78.7	76.9	NA	67.5	69.4
After	27.9	3.6	NA	63.8	61.6
Concurrent use of cilostazol 200 mg and potent CYP3A4 or CYP2C19 inhibitors (%)^f^					
Before	19.6	10.0	NA	2.1	3.6
After	5.8	0.0	NA	0.7	1.9

The terms *before* and *after* refer to the periods before and after the implementation of risk minimization periods.

CI, confidence interval; EpiChron, EpiChron cohort from Aragon Health Sciences Institute (IACS); GePaRD, German Pharmacoepidemiological Research Database; NA, not available; RR, rate ratio; SIDIAP, Information System for the Improvement of Research in Primary Care; THIN, The Health Improvement Network; UK, United Kingdom.

a Current smoking at the start date. In Sweden, smoking was evaluated only through smoking‐related diagnoses and dispensations for smoking‐cessation drugs.

b Percentage of users with at least 1 visit to a specialist (vascular surgery, cardiology, diabetology) 2 to 4 months after the start date.

c Discontinuation of cilostazol within the first 3 months of treatment.

d Unstable angina pectoris and myocardial infarction or coronary intervention within the last 6 months.

e Rate ratio of visits to the general practitioner or specialist between users with and without increased risk of serious cardiac events (arrhythmias, hypotension, or coronary heart disease). In GePaRD, visits were expressed as the number of diagnoses per patient‐year of continuous use, because only the first visit to the same physician is recorded during a quarter.

f Potent CYP3A4 or CYP2C19 inhibitors: lansoprazole, fluvoxamine, nefazodone, ticlopidine, clarithromycin, troleandomycin, indinavir, ritonavir, nelfinavir, mibefradil, ketoconazole, and itraconazole.

## 4 DISCUSSION

In this study, we evaluated the effectiveness of risk minimization measures for the use of cilostazol in the UK, Spain, Sweden, and Germany. The study addressed the concerns raised during the EMA Article 31 cilostazol referral and the requirement to evaluate the risk minimization measures through drug utilization studies. The characteristics of new users of cilostazol remained similar before and after implementation of risk minimization activities. In both periods, there was a higher proportion of men than women, and most users were elderly and had a high prevalence of comorbidity, especially cardiovascular disease, and concurrent use of other medications. In general, the risk minimization measures were effective in all study populations, as indicated by the marked decrease in the prevalence of cilostazol use, the decrease of use in the presence of new cardiovascular contraindications, and the lower concurrent use of cilostazol and interacting medications, including CYP2C19 and CYP3A4 potent inhibitors. Early monitoring and discontinuation of cilostazol, concurrent use with 2 or more antiplatelet agents, monitoring of users at high risk of cardiovascular events, and the prevalence of old contraindications improved in most study populations after labeling changes. Current smoking at the initiation of cilostazol improved only in EpiChron, in Spain. The prevalence of use of cilostazol started diminishing in some countries while regulatory reviews were ongoing and before actual implementation of the labeling changes. Prevalence of use continued decreasing until 2014, after implementation of risk minimization measures. The decrease is consistent with the reduction of cilostazol sales in the study countries and Europe overall, according to data provided by Otsuka, the manufacturer of cilostazol. The characteristics of users of cilostazol in this study are in line with those from a study conducted in Spain.^15^ In that study, most users were elderly and had a high prevalence of comorbidity and use of comedications.

A strength of our study is the use of automated health databases, allowing evaluation of medication use as prescribed in routine health care without interfering with or modifying clinical practice. The relation between the labeling changes and the variables used to measure them is of great relevance to the interpretation of the study. Choice of measures was challenging given the data available across data sources. For some labeling changes, there was no information; only proxies or partial data could be used. Early in the design discussions, considerable time was devoted to this aspect, and whether de novo data collection via prescriber questionnaires would be more informative. In the end, given the challenges of the latter approach, including potential self‐selection bias of participating health care practitioners, we selected the database approach as the best means to evaluate the impact of the labeling changes overall. This permitted us to evaluate the effectiveness of the cilostazol risk minimization measures in different countries and health systems.

Several considerations should be considered when reviewing our results. As mentioned previously, information for some labeling changes (eg, physician reassessment of treatment) was not available in the study databases. The use of proxies for these items could lead to some misclassification of the actual labeling change before and after implementation of risk minimization measures. Also, as in any before‐after study without a comparator mediation, factors other than the risk minimization measures could have influenced the changes observed in the conditions included in the new labeling of cilostazol. The EMA cilostazol referral itself could affect the prescribing of cilostazol, as physicians' and prescribers' awareness of the potential safety issues may have increased during the referral period, before the risk minimization measures were implemented. In fact, the decreasing prevalence of cilostazol use beginning in 2011 to 2012, before implementation of risk minimization measures in some databases, is consistent with an effect of the referral process itself.

The period before implementation of risk minimization measures included many users in most study populations as the study periods covered several years: approximately 3.5 years in EpiChron and SIDIAP and 10 years in THIN. However, the period after the risk minimization measures was restricted to new users identified during 2014. Therefore, the “after” period included only a small number of users in all databases, with a shorter time of follow‐up, increasing random variability and potential underascertainment of variables measured during cilostazol use in the follow‐up period. Also, restricting the period after implementation of risk‐minimization measures to 1 year did not allow assessment of the long‐term effectiveness of these measures. Long‐term low compliance of lifestyle recommendations among patients with peripheral arterial disease has been reported.^16^ Evaluation of cilostazol users before implementation of risk minimization measures reflects the average profile of users through a long period and not the actual profile immediately before risk minimization activities were implemented. Characteristics of users and patterns of use could have changed since cilostazol became available for the treatment of intermittent claudication; patients and health care practitioners in 2012 may have been more aware of potential problems than those initiating cilostazol in 2002. However, we believe the concern is not great because it does not impact the Spanish data sources, which provided the largest number of users. In the United Kingdom, a small monotonic increase in the prevalence of users during the research period was observed from 2003 to 2011, and use of cilostazol increased in Germany from 2007 to 2011. Neither situation suggests a strong awareness of potential problems with cilostazol. In Sweden, a small decrease in the prevalence of use of cilostazol occurred in the last 3 years; however, we have data for only 4 years, and the prevalence of users of cilostazol in 2012 (13 per 100 000) is equivalent to that in 2009 (13 per 100 000) and not very different from the maximum prevalence (16.5 per 100 000 in 2010), limiting our concern about lack of comparability.

Changes in the recording of diagnoses in the study databases before and after the risk minimization measures could affect the comparison of results between the 2 periods. For example, after labeling changes, the recording of the ankle‐brachial index was implemented in SIDIAP, resulting in a higher prevalence of diagnoses. In addition, clinical guidelines and the introduction of new and generic medications in the period after labeling changes also need to be considered. For example, direct oral anticoagulants were introduced recently in most countries; health services in Catalonia (SIDIAP) tried to reduce the consumption of proton pump inhibitors and encouraged general practitioners to review patient prescriptions periodically, and atorvastatin became generic in Germany.

Differences in the type of databases included in this study could introduce variability in the baseline prevalence of conditions across the study populations before and after implementation of risk minimization measures. Thus, information recorded in THIN, EpiChron, and SIDIAP was based on primary care electronic medical records, information recorded in Sweden on inpatient and outpatient hospital discharge diagnoses, and information recorded in GePaRD on insurance claims from ambulatory care and hospital admissions. This impacted the evaluation of some variables such as smoking status at the start date, which in Sweden was ascertained indirectly using diagnosis codes related to smoking‐related illnesses, and the prevalence was underestimated. History of smoking‐related diagnoses could be a poor marker of current smoking, leading to misclassification. Although differences between databases limited some baseline comparisons, within‐database comparisons, before and after risk minimization, provided an efficient framework to evaluate the impact of risk minimization measures in different countries and health systems.

Results from this study can be generalized to each respective country. The THIN database includes information for approximately 6% of the UK population, and the population covered in the database has been shown to have demographics, deprivation index, disease prevalence, and mortality rates similar to the overall UK population.^3^ In Spain, EpiChron covers all the primary care practices of the public health system in the region of Aragon, and SIDIAP covers about 80% of those in the region of Catalonia.^17^, ^18^ In Sweden, data included in the study involve the entire population. In Germany, the data covered approximately 10% of the German population, about 8.4 million and 8.0 million insured members before and after implementation of risk minimization measures, respectively.

Overall, results from this study conducted in the United Kingdom, Spain, Sweden, and Germany are compatible with a positive effect of implementing risk minimization measures in all the study populations, as indicated by the lower prevalence of cilostazol use and the improvement of most utilization parameters evaluated. However, the risk minimization measures impacted the prevalence of smoking at the time of initiating cilostazol treatment in only 1 of the 4 study populations where smoking was evaluated. These findings should be interpreted with caution given the random variation introduced by the small number of new users of cilostazol and the short time of follow‐up after implementation of risk minimization measures.

## CONFLICT OF INTEREST

- Susana Perez‐Gutthann, Brian Calingaert, Christine Bui, and Alejandro Arana are employees of RTI Health Solutions and work on projects funded by pharmaceutical companies. As an employee of RTI Health Solutions, Susana Perez‐Gutthann also participates in scientific advisory boards (for studies and medications) that are funded by pharmaceutical companies. Jordi Castellsague was a full‐time employee of RTI Health Solutions at the time of the study.
- Alexandra Prados‐Torres, Beatriz Poblador‐Plou, Francisca Gonzalez‐Rubio, and Clara Laguna are members of the EpiChron Research Group on Chronic Diseases of the Aragon Health Sciences Institute (IACS), ascribed to IIS Aragon, and do not have any conflict of interest with this project.
- Maria Giner‐Soriano and Albert Roso‐Llorach, as employees of IDIAP Jordi Gol, worked on other projects funded by pharmaceutical companies in their institution that were not related to this study and without personal profit.
- Marie Linder is employed at the Centre for Pharmacoepidemiology, Karolinska Institutet, which receives grants from several entities (pharmaceutical companies, regulatory authorities, and contract research organizations) for performance of drug safety and drug utilization studies.
- Oliver Scholle is an employee of the nonprofit scientific organization Leibniz Institute for Prevention Research and Epidemiology—BIPS, which is, among others, conducting studies financed by pharmaceutical companies based on data provided by German statutory health insurance agencies.

## SOURCES OF SUPPORT

The study was funded by Otsuka Pharmaceutical Europe Ltd., Gallions, Wexham Springs Framewood Road, Wexham, SL3 6PJ, United Kingdom. The contract provides the research team independent publication rights. The sponsor had no role in the data collection or analysis and was not involved in the interpretation of results or the decision to submit the manuscript; however, in line with the *Guideline on Good Pharmacovigilance Practices (GVP): Module VIII‐Post‐authorisation Safety Studies* of the European Medicines Agency, the sponsor had the opportunity to view the results and interpretations included in the manuscript and provide comments prior to submission of the manuscript for publication.

## Supporting information

Table S1. Main Features of the Study Databases
Table S2. Most Frequent Comorbidities (%) Among New Users of Cilostazol Before and After the Implementation of Risk Minimization Measures
Table S3. Most Frequent Comedications (%) Among New Users of Cilostazol Before and After the Implementation of Risk Minimization Measures
Table S4. Concurrent Use (%) of Most Frequently Used Potentially Interacting Medications Before and After the Implementation of Risk Minimization Measures
Table S5. Assessment of Old Contraindications Before and After the Implementation of Risk Minimization Measures
