# Supplementary material for: Effectiveness of risk minimization measures for the use of cilostazol in United Kingdom, Spain, Sweden, and Germany
Source: Pharmacoepidemiol Drug Saf. 2018 Jul 25;27(9):953–61. doi: 10.1002/pds.4584 (PMC6175151; doi:10.1002/pds.4584)
Supplement: Supplementary file 1 — Table S1. Main Features of the Study Databases Table S2. Most Frequent Comorbidities (%) Among New Users of Cilostazol Before and After the Implementation of Risk Minimization Measures Table S3. Most Frequent Comedications (%) Among New Users of Cilostazol Before and After the Implementation of Risk Minimization Measures Table S4. Concurrent Use (%) of Most Frequently Used Potentially Interacting Medications Before and After the Implementation of Risk Minimization Measures Table S5. Assessment of Old Contraindications Before and After the Implementation of Risk Minimization Measures [file PDS-27-953-s001.docx]

## ONLINE SUPPORTING INFORMATION

Table S1. Main Features of the Study Databases

| Feature | THIN, United Kingdom | EpiChron Cohort, Aragon, Spain | SIDIAP, Catalonia, Spain | Swedish National Prescription and Inpatient Registers | GePaRD, Germany |
| --- | --- | --- | --- | --- | --- |
| Country/region population | 63,022,532^†^ | 1,344,508^‡^ | 7,508,106^§^ | 9,415,570^†^ | 80,222,065^†^ |
| Database population | 3.6 million | 1.3 million | 5.8 million | 9.7 million | 17 million |
| Database type | Primary health care electronic medical record database | Primary health care electronic medical record database; link to community pharmacy data | Primary health care electronic medical record database; link to hospital discharge and community pharmacy data | National health record databases linked through the unique civil personal registration number | Claims databases; four SHI providers^¶^ |
| Primary care diagnosis | Yes | Yes | Yes | Outpatient hospital clinics | Yes |
| Hospital diagnosis | Recorded by GPs | No | Yes | Yes | Yes |
| Disease codes | Read codes | ICPC-2 | ICD-10-CM | ICD-10-CM | ICD-10-GM |
| Procedures codes | Read codes | Not available | Not available | NOMESCO | OPS and EBM |
| Lifestyle risk factors | Yes | No | Yes | No | No |
| Socioeconomic status | Townsend deprivation index^††^ | Not available | Medea deprivation index^‡‡^ | Family income; number of years of education | Not available |
| Medications | Prescriptions written by GPs | Reimbursed pharmacy-dispensed prescriptions | Reimbursed pharmacy-dispensed prescriptions written in primary care centers | All pharmacy-dispensed prescriptions | Reimbursed pharmacy-dispensed prescriptions |
| Dose | Prescribed dose | Formulation strength | Formulation strength | Formulation strength | Formulation strength |
| Duration | Prescribed duration | Not recorded | Not recorded | Not recorded | Not recorded |
| Medication codes | Multilex/British National Formulary | ATC | ATC | ATC | ATC |

ATC = Anatomical Therapeutic Chemical classification; EBM = Einheitlicher Bewertungsmaßstab; EpiChron = EpiChron cohort from Aragon Health Sciences Institute (IACS), Aragón, Spain; GePaRD = German Pharmacoepidemiological Research Database; GP = general practitioner; ICD‑10‑CM = *International Statistical Classification of Diseases and Related Health Problems, 10th Revision, Clinical Modification*; ICD‑10-GM = ICD-10, German Modification; ICPC-2 = International Classification of Primary Care, Second Edition; NOMESCO = Classification of Surgical Procedures, Version 1.16; OPS = Operationen- und Prozedurenschlüssel; SHI = Statutory Health Insurance; SIDIAP = Information System for the Advancement of Research in Primary Care; THIN = The Health Improvement Network Database.

^†^ European population from Eurostat. 2011. Available at: <http://ec.europa.eu/eurostat/tgm/table.do?tab=table&init=1&language=en&pcode=tps00001&plugin=1>. Accessed 26 October 2017.

^‡^ Government of Aragón. Available at: <http://www.aragon.es/estaticos/GobiernoAragon/Organismos/InstitutoAragonesEstadistica/Documentos/docs/Areas/Demogra/6.-%20CensoPoblacionViviendas/2011/fichas/A02.pdf>. Accessed 26 October 2017.

^§^ Generalitat de Catalunya. Available at: <http://web.gencat.cat/ca/temes/catalunya/coneixer/>. Accessed 26 October 2017.

^¶^ Two SHIs covering approximately 9 million population contributed data for this study.

^††^ Townsend P, Phillimore P, Beattie A. Health and deprivation: inequality and the north. London: Croom Helm; 1988.

^‡‡^ Domínguez-Berjón M, Borrell C, Cano-Serral G, Esnaola S, Nolasco A, Pasarín M, et al. Construcción de un índice de privación a partir de datos censales en grandes ciudades españolas (Proyecto MEDEA). Gac Sanit 2008;22(3):179-87.

Table S2. Most Frequent Comorbidities (%) Among New Users of Cilostazol Before and After the Implementation of Risk Minimization Measures

|  | Risk Minimization Period | THIN UK | EpiChron Aragón Spain | SIDIAP Catalonia Spain | Sweden | GePaRD Germany |
| --- | --- | --- | --- | --- | --- | --- |
| Number of users | Before | 1,528 | 4,024 | 10,142 | 2,887 | 4,012 |
|  | After | 104 | 367 | 771 | 149 | 430 |
| Comorbidity |  |  |  |  |  |  |
| Cardiovascular disease† | Before | 75.7 | 74.5 | 82.2 | 62.8 | 95.7 |
|  | After | 76.0 | 57.8 | 83.9 | 63.8 | 95.3 |
| Peripheral arterial disease | Before | 72.1 | 36.1 | 50.3 | 55.6 | 92.0 |
|  | After | 64.4 | 48.8 | 79.2 | 38.9 | 93.7 |
| Hypertension | Before | 54.0 | 54.9 | 63.0 | 46.8 | 86.0 |
|  | After | 53.8 | 39.2 | 64.9 | 53.7 | 87.9 |
| Ischemic heart disease | Before | 32.5 | 14.0 | 17.2 | 31.6 | 52.6 |
|  | After | 25.0 | 9.5 | 12.3 | 28.9 | 52.1 |
| Hyperlipidemia | Before | 31.3 | 37.4 | 48.5 | 20.4 | 75.3 |
|  | After | 36.5 | 39.5 | 56.4 | 20.8 | 80.9 |
| Skin disorders | Before | 26.1 | 15.9 | 8.7 | 7.8 | 42.1 |
|  | After | 36.5 | 9.8 | 16.3 | 12.8 | 54.9 |
| Renal disease | Before | 27.5 | 12.8 | 16.4 | 15.8 | 48.1 |
|  | After | 31.7 | 5.2 | 25.8 | 15.4 | 55.8 |
| Bleeding disorders | Before | 22.6 | 4.0 | 5.6 | 11.7 | 27.9 |
|  | After | 30.8 | 3.3 | 9.7 | 15.4 | 34.9 |
| Diabetes mellitus | Before | 21.3 | 29.9 | 40.4 | 20.5 | 41.1 |
|  | After | 20.2 | 23.4 | 40.3 | 20.1 | 39.1 |

EpiChron, EpiChron cohort from Aragon Health Sciences Institute (IACS); GePaRD, German Pharmacoepidemiological Research Database; SIDIAP, Information System for the Improvement of Research in Primary Care database; THIN, The Health Improvement Network; UK, United Kingdom.

† Cardiovascular diseases: hypertension, ischemic heart disease, hyperlipidemia, cerebrovascular diseases, arrhythmias, heart failure, hypotension, conduction disorders, cardiac arrest, and other cardiovascular diseases. Excludes peripheral arterial disease.

Table S3. Most Frequent Comedications (%) Among New Users of Cilostazol Before and After the Implementation of Risk Minimization Measures

|  | Risk Minimization Period | THIN UK | EpiChron Aragón Spain | SIDIAP Catalonia Spain | Sweden | GePaRD Germany |
| --- | --- | --- | --- | --- | --- | --- |
| Number of users | Before | 1,528 | 4,024 | 10,142 | 2,887 | 4,012 |
|  | After | 104 | 367 | 771 | 149 | 430 |
| Comedication |  |  |  |  |  |  |
| Antihypertensives† | Before | 71.5 | 63.6 | 74.5 | 80.6 | 77.7 |
|  | After | 65.4 | 64.3 | 68.5 | 70.5 | 77.7 |
| Lipid-modifying agents‡ | Before | 68.6 | 45.8 | 63.6 | 61.6 | 48.8 |
|  | After | 75.0 | 54.5 | 64.9 | 52.3 | 52.6 |
| Platelet aggregation inhibitors§ | Before | 67.3 | 46.9 | 73.1 | 69.7 | 33.8 |
|  | After | 59.6 | 54.2 | 80.0 | 59.1 | 34.4 |
| Statins | Before | 66.8 | 41.8 | 60.3 | 60.3 | 42.9 |
|  | After | 73.1 | 49.6 | 62.4 | 51.7 | 49.3 |
| Renin-angiotensin system agents | Before | 48.8 | 49.9 | 61.7 | 54.7 | 62.8 |
|  | After | 47.1 | 55.3 | 58.5 | 51.0 | 63.0 |
| Calcium channel blockers | Before | 34.5 | 18.6 | 23.6 | 37.8 | 25.3 |
|  | After | 31.7 | 18.8 | 20.1 | 31.5 | 27.4 |
| Diuretics | Before | 33.2 | 20.9 | 26.4 | 33.7 | 25.0 |
|  | After | 24.0 | 15.5 | 21.1 | 21.5 | 21.9 |
| Proton pump inhibitors | Before | 30.0 | 53.2 | 60.9 | 22.4 | 25.0 |
|  | After | 49.0 | 50.7 | 49.7 | 26.2 | 32.6 |
| Musculoskeletal system drugs | Before | 24.5 | 34.3 | 39.0 | 19.4 | 29.7 |
|  | After | 14.4 | 29.4 | 19.8 | 12.1 | 29.3 |
| Beta-blocking agents | Before | 22.2 | 14.7 | 18.4 | 44.1 | 45.6 |
|  | After | 31.7 | 16.6 | 16.2 | 38.3 | 46.0 |
| Peripheral vasodilators | Before | 12.5 | 33.6 | 37.7 | 0.3 | 11.7 |
|  | After | 57.7 | 32.4 | 19.7 | 0.7 | 1.4 |
| Blood glucose–lowering drugs | Before | 13.8 | 20.9 | 32.2 | 16.4 | 18.6 |
|  | After | 10.6 | 26.7 | 31.9 | 16.1 | 13.7 |

EpiChron, EpiChron cohort from Aragon Health Sciences Institute (IACS); GePaRD, German Pharmacoepidemiological Research Database; SIDIAP, Information System for the Improvement of Research in Primary Care database; THIN, The Health Improvement Network; UK, United Kingdom.

† Antihypertensives: renin-angiotensin system agents, calcium channel blockers, diuretics, beta-blocking agents, and other antihypertensives (antiadrenergic agents, agents acting on arteriolar smooth muscle, antihypertensives and diuretics in combination, and other antihypertensives and combinations).

‡ Lipid-modifying agents: statins, fibrates, bile acid sequestrants, nicotinic acid and derivatives, and other lipid-modifying agents.

§ Platelet aggregation inhibitors, excluding cilostazol.

Table S4. Concurrent Use (%) of Most Frequently Used Potentially Interacting Medications Before and After the Implementation of Risk Minimization Measures

|  | Risk Minimization Period | THIN UK | EpiChron Aragón Spain | SIDIAP Catalonia Spain | Sweden | GePaRD Germany |
| --- | --- | --- | --- | --- | --- | --- |
| Number of users | Before | 1,528 | 4,024 | 10,142 | 2,887 | 4,012 |
|  | After | 104 | 367 | 771 | 149 | 430 |
| Potentially interacting medication |  |  |  |  |  |  |
| Any potentially interacting medication | Before | 91.6 | 82.5 | 90.0 | 84.4 | 78.8 |
|  | After | 91.3 | 79.0 | 84.7 | 79.9 | 81.4 |
| Any potent inhibitors of CYP3A4 or CYP2C19 enzymes | Before | 22.3 | 10.2 | 7.3 | 2.7 | 3.8 |
|  | After | 17.3 | 3.0 | 2.1 | 0.7 | 2.3 |
| Medications interacting with CYP3A4 (any) | Before | 85.3 | 57.2 | 73.2 | 78.2 | 66.0 |
|  | After | 83.7 | 55.6 | 70.9 | 71.1 | 70.7 |
| Simvastatin | Before | 44.0 | 17.6 | 38.0 | 55.7 | 48.9 |
|  | After | 42.3 | 21.5 | 39.2 | 38.9 | 41.2 |
| Atorvastatin | Before | 29.3 | 26.8 | 26.7 | 10.1 | 0.9 |
|  | After | 26.0 | 24.8 | 24.5 | 23.5 | 14.7 |
| Amlodipine | Before | 22.2 | 7.7 | 16.7 | 18.5 | 19.8 |
|  | After | 13.5 | 7.6 | 15.4 | 21.5 | 22.3 |
| Medications interacting with CYP2C19 (any) | Before | 55.3 | 71.7 | 75.3 | 37.4 | 47.8 |
|  | After | 58.7 | 61.3 | 58.6 | 30.9 | 48.6 |
| Omeprazole | Before | 22.4 | 47.7 | 59.3 | 23.6 | 17.2 |
|  | After | 31.7 | 42.5 | 47.6 | 18.8 | 10.0 |
| Clopidogrel | Before | 18.2 | 23.4 | 22.5 | 11.7 | 21.4 |
|  | After | 11.5 | 17.2 | 11.2 | 7.4 | 18.8 |

EpiChron, EpiChron cohort from Aragon Health Sciences Institute (IACS); GePaRD, German Pharmacoepidemiological Research Database; SIDIAP, Information System for the Improvement of Research in Primary Care database; THIN, The Health Improvement Network; UK, United Kingdom.

**Table S5. Assessment of Old Contraindications Before and After the Implementation of Risk Minimization Measures**

| By Risk Minimization Period | THIN UK | EpiChron Aragón Spain | SIDIAP Catalonia Spain | Sweden | GePaRD Germany |
| --- | --- | --- | --- | --- | --- |
| Number of users |  |  |  |  |  |
| Before | 1,528 | 4,024 | 10,142 | 2,887 | 4,012 |
| After | 104 | 367 | 771 | 149 | 430 |
| Contraindication |  |  |  |  |  |
| Any contraindication (%) |  |  |  |  |  |
| Before | 10.0 | 6.2 | 39.1 | 12.2 | 51.8 |
| After | 8.7 | 5.5 | 51.5 | 12.1 | 54.7 |
| Renal failure (%) |  |  |  |  |  |
| Before | 2.4 | 0.0 | 7.9 | 2.8 | 20.7 |
| After | 4.8 | 0.0 | 13.0 | 3.4 | 27.0 |
| Liver disease (%) |  |  |  |  |  |
| Before | 1.3 | 1.6 | 3.7 | 1.0 | 25.4 |
| After | 0.0 | 2.7 | 6.9 | 1.3 | 24.2 |
| Heart failure (%) |  |  |  |  |  |
| Before | 4.8 | 2.9 | 3.7 | 3.0 | 3.9 |
| After | 2.9 | 1.6 | 3.8 | 3.4 | 4.0 |
| Conditions predisposing to bleeding (%) |  |  |  |  |  |
| Before | 1.8 | 1.7 | 29.9 | 5.7 | 16.3 |
| After | 1.0 | 1.4 | 38.5 | 6.0 | 16.0 |
| Active peptic ulcer (%) |  |  |  |  |  |
| Before | 0.1 | 0.1 | 0.1 | 0.4 | 3.9 |
| After | 0.0 | 0.0 | 0.4 | 0.7 | 3.5 |
| Recent cerebral hemorrhage (%)† |  |  |  |  |  |
| Before | 0.0 | 0.0 | 0.2 | 0.1 | 0.6 |
| After | 0.0 | 0.0 | 0.0 | 0.0 | 0.2 |
| Proliferative diabetic retinopathy (%)‡ |  |  |  |  |  |
| Before | 0.7 | 1.7 | 4.5 | 5.2 | 12.4 |
| After | 0.0 | 1.4 | 7.5 | 5.4 | 12.6 |
| Poorly controlled hypertension (%) |  |  |  |  |  |
| Before | 1.0 | NA | 26.6 | NA | NA |
| After | 1.0 | NA | 34.1 | NA | NA |
| Arrhythmias (%)§ |  |  |  |  |  |
| Before | 0.7 | 0.2 | 5.9 | 1.4 | 8.3 |
| After | 0.0 | 0.0 | 0.1 | 0.7 | 9.5 |

EpiChron, EpiChron cohort from Aragon Health Sciences Institute (IACS); GePaRD, German Pharmacoepidemiological Research Database, Germany; NA, not available; SIDIAP, Information System for the Improvement of Research in Primary Care; THIN, The Health Improvement Network, UK; UK, United Kingdom.

Note: Old contraindications refer to contraindications that were in the cilostazol labelling before the implementation of labelling changes in 2013. The terms “Before” and “After” refer to the period before and after the implementation of risk minimization measures.

† Hemorrhagic stroke within the prior 6 months.

‡ Assessed with codes for diabetic retinopathy in EpiChron, Sweden, and Germany.

§ Ventricular tachycardia, ventricular fibrillation or multifocal ventricular ectopic beats, and QT interval prolongation. In EpiChron, only paroxysmal ventricular tachycardia was evaluated.
